# Analysis of spinal muscular atrophy carrier screening results in 32,416 pregnant women and 7,231 prepregnant women

**DOI:** 10.3389/fneur.2024.1357476

**Published:** 2024-04-09

**Authors:** Bing-bo Zhou, Xue Chen, Chuan Zhang, Yu-pei Wang, Pan-pan Ma, Sheng-ju Hao, Ling Hui, Yun-fei Bai

**Affiliations:** ^1^State Key Laboratory of Digital Medical Engineering, School of Biological Science and Medical Engineering, Southeast University, Nanjing, China; ^2^The Center for Medical Genetics in Gansu Provincial Maternity and Child-care Hospital, Gansu Provincial Clinical Research Center for Birth Defects and Rare Diseases, Lanzhou, China

**Keywords:** SMA carrier screening, genetics counseling, *SMN1*, pre-pregnancy, pregnancy

## Abstract

**Objectives:**

Spinal muscular atrophy (SMA) is an autosomal recessive disease that is one of the most common in childhood neuromuscular disorders. Our screenings are more meaningful programs in preventing birth defects, providing a significant resource for healthcare professionals, genetic counselors, and policymakers involved in designing strategies to prevent and manage SMA.

**Method:**

We screened 39,647 participants from 2020 to the present by quantitative real-time PCR, including 7,231 pre-pregnancy participants and 32,416 pregnancy participants, to detect the presence of SMN1 gene EX7 and EX8 deletion in the DNA samples provided by the subjects. To validate the accuracy of our findings, we also utilized the Multiplex Ligation-dependent Probe Amplification (MLPA) to confirm the reliability of screening results obtained by quantitative real-time PCR.

**Result:**

Among the 39,647 participants who were screened, 726 participants were the carriers of *SMN1*. The overall carrier rate was calculated to be 1.83% (95% confidence interval: 0.86–2.8%). After undergoing screening, a total of 592 pregnancy carriers were provided with genetic counseling and only 503 of their spouses (84.97, 95% confidence interval: 82.09–87.85%) voluntarily underwent SMA screening.

**Conclusion:**

This study provides crucial insights into the prevalence and distribution of SMA carriers among the female population. The identification of 726 asymptomatic carriers highlights the necessity of comprehensive screening programs to identify at-risk individuals and ensure appropriate interventions are in place to minimize the impact of SMA-related conditions.

## 1 Introduction

Spinal muscular atrophy (SMA) is one of the most common neuromuscular diseases in children, with muscle weakness and muscle atrophy caused by the defects of α-motor neurons in the anterior horn of the spinal cord as the main clinical features. In 1995, scientists made a significant breakthrough by identifying a survival motor neuron (*SMN1*) gene which is located in the chromosomal region of 5q11.2-q13.3 (1), as the culprit for SMA, an autosomal recessive disorder. *SMN1* is responsible for encoding the vital survival motor neuron protein in motor neurons, thus playing a crucial role in maintaining their proper functioning (2). SMA is identified as the second most common inherited cause of infant mortality, with an estimated incidence of 1/6000 ~ 1/11000 live births, and the carrier frequency is 1/47 ~ 1/72 (3, 4).

SMA is a disease that manifests itself in varying degrees of severity, and patients can exhibit a wide spectrum of clinical symptoms. To classify the disease and its various manifestations, clinicians generally divide SMA into four distinct clinical types based on their diagnostic criteria (5): (1) Type I is the most severe and common type, which accounts for about 50% of patients diagnosed with SMA, and is usually characterized by an inability to walk unaided, weakness in the neck, and an inability to care for oneself. (2) Type II is relatively mild compared to Type I. Patients can sit without support and in some cases, they may even be able to stand up, but they do not gain the ability to walk on their own. (3) Type III is relatively milder compared to Type II. Patients with this condition may exhibit clinical heterogeneity, meaning they can present with varying symptoms and severity. However, most patients with type III exhibit muscle atrophy and loss of ambulation. (4) Type IV is characterized by mild symptoms and typically appears after the age of 30. Patients with Type IV are usually able to walk without any issues related to breathing or eating (4, 6). Approximately 95% of SMA patients are caused by loss of function in the *SMN1* due to E7 lost or both E7 and E8 lost, while the remaining individuals may exhibit a genetic variation where one copy of the gene is lost and the other copy contains a mutation within the gene (7). The disease severity is mainly influenced by the copy number of survival motor neuron 2 (*SMN2*), a nearly identical gene and highly similar to *SMN1*, which produces only less functional protein (8). The *SMN1* and *SMN2* are very similar in terms of their DNA sequences, with only five nucleotides differentiating them. Of these nucleotides, the four nucleotides are located in the non-coding region (2), and a special site (c.840C > T) in the coding region is used to distinguish *SMN1* from*SMN2* in the next-generation sequencing technology (9).

Considering the severity of clinical symptoms, high mortality rate, and strong genotype–phenotype correlation, for a long time, screening for SMA carriers has been of interest to clinicians. In 2008, the American College of Medical Genetics (ACMG) recommended that individuals be screened for SMA carrier status regardless of their race or ethnicity. This population screening is intended to identify carriers of the SMA gene, which can help with family planning and early detection of symptoms in affected children. By identifying carriers in the general population, healthcare providers can better inform individuals and couples about the risks of passing on the SMA gene to their children. This recommendation reflects the importance of early detection and prevention of genetic diseases like SMA (10). In 2016, the American College of Obstetrics and Gynecology (ACOG) recommended that all women who are planning to become pregnant or are already pregnant should undergo screening for SMA carriers. This recommendation was made to identify carriers of SMA, a genetic disorder that can be passed on to offspring, and to provide appropriate counseling and care to affected families. Therefore, it is important for women to be aware of this recommendation and to discuss SMA carrier screening with their healthcare provider (11). The therapeutic medication for SMA, nusinersen, has been available in mainland China since 2019, and an expert consensus on genetic diagnosis of SMA has been published (12, 13). Through meticulous and thorough screening, it is feasible to detect people who possess *SMN1* mutations in a heterozygous condition, which denotes that they are carriers of SMA. If both partners of a couple are SMA carriers, there is a 25% possibility that their offspring may inherit SMA. It is advisable to undergo prenatal diagnosis of SMA with standard genetic counseling. Currently, most countries and regions, including some parts of China, routinely screen parents and newborns for SMA mutations (14–19). In this study, we report our SMA carrier screening data for >30,000 individuals without a family history of SMA in Lanzhou City, Gansu Province, Northwest China, which was performed using technical criteria and guidelines for SMA testing and conducted using real-time quantitative polymerase chain reaction (real-time PCR, or qPCR). Our findings highlight the significant impact of the ACMG guidelines, which led to an increase in the adoption of rapid testing and the identification of SMA carriers in the area. Additionally, this study sheds light on the high level of patient interest in screening for SMA carriers. Furthermore, the availability of prenatal diagnosis for high-risk patients can effectively prevent the birth of a child with SMA.

## 2 Materials and methods

### 2.1 Population samples and processing flow

This clinical study was performed at the Center of Genetics of Gansu provincial Maternity and Child-care Hospital between Jan 1, 2020, and Mar 31, 2023. A total of 39,647 women were accepted to SMA carrier screening, including 32,416 pregnancy participants (Supplementary Figure S1; Table 1) and 7,231 pre-pregnancy participants (Supplementary Figure S1; Table 2). Before the screening program, they were all received genetic counseling. Information on the participants is listed in Tables 1, 2. The ages of the participants during the pre-pregnancy and pregnancy screening mainly ranged from 25 to 35 years and the gestational weeks of the pregnancy screening participants mainly ranged from 12 to 14 weeks (Supplementary Figure S2). If a woman was a carrier of SMA, then her spouse needed to be screened. For couples who were SMA carriers, the fetus was carried out SMA prenatal diagnosis according to the wishes of both couples. The screening is limited to the deletion of *SMN1* (exon7/exon8), and other genes are not in the detection range. This study was approved by the ethics committee of Gansu provincial Maternity and Child-care Hospital. All participants signed written informed consent.

**Table 1:** Details of the 32,416 pregnant women in different time periods.

Screening Date	2020.3–2020.12			2021.1–2021.6			2021.7–2021.12			2022.1–2022.6			2022.7–2022.12			2023.1–2023.4		
Total Number	4,200			4,474			6,658			5,657			6,352			5,075		
Ages (Years)	*N*	%	95% CI	*N*	%	95% CI	*N*	%	95% CI	*N*	%	95% CI	*N*	%	95% CI	*N*	%	95% CI
<25	471	11.21	8.36–14.06	523	11.69	8.94–14.44	804	12.08	9.82–14.33	605	10.69	8.23–13.16	580	9.13	6.79–11.48	488	9.62	7–12.23
26–35	3,290	78.33	76.93–79.74	3,622	80.96	79.68–82.24	5,399	81.09	80.05–82.13	4,666	82.48	81.39–83.57	5,288	83.25	82.24–84.26	4,128	81.34	80.15–82.53
>35	439	10.45	7.59–13.31	329	7.35	4.53–10.17	455	6.83	4.52–9.15	386	6.82	4.31–9.34	484	7.62	5.26–9.98	459	9.04	6.42–11.67
Gestational age (Weeks)																		
<15	3,218	76.62	75.16–78.08	3,726	81.96	80.72–83.21	5,501	82.62	81.62–83.62	4,722	83.47	82.41–84.53	5,203	81.91	80.87–82.96	4,072	80.24	79.01–81.46
16–21	853	20.31	17.61–23.01	688	15.38	12.68–18.07	1,022	15.35	13.14–17.56	802	14.18	11.76–16.59	976	15.37	13.1–17.63	767	15.11	12.58–17.65
>21	129	3.07	0.09–6.05	119	2.66	−5.78	135	2.03	−4.76	133	2.35	−5.15	173	2.72	0.3–5.15	236	4.65	1.96–7.34

**Table 2:** Details of the 7,231 pre-pregnancy women in different time periods.

Screening date	2020			2021			2022			2023		
Total number	619			2,859			2,662			1,084		
Ages (Years)	*N*	%	95% CI	*N*	%	95% CI	*N*	%	95% CI	*N*	%	95% CI
<25	81	13.09	5.74–20.43	335	11.72	8.27–15.16	240	9.02	5.39–12.64	105	9.69	4.03–15.34
26–35	479	77.38	73.64–81.13	2,248	78.63	76.93–80.32	2,158	81.07	79.41–82.72	848	78.23	75.45–81.01
>35	59	9.53	2.04–17.02	276	9.65	6.17–13.14	264	9.92	6.31–13.52	131	12.08	6.5–17.67
Number of carriers (*n*)	14			46			47			27		
Carrier rate (%)	2.26			1.61			1.76			2.49		
95% CI (%)	1.09–3.43			1.15–2.07			1.26–2.26			1.56–3.42		
Spouse (*n*)	13			40			42			25		
Recall rate (%)	92.86			86.95			89.36			92.59		
95% CI (%)	79.37–106.35			77.22–96.68			80.54–98.18			82.71–102.47		
Number of carrier couples	1			1			1			0		

### 2.2 Genomic DNA

A total of 2 ~ 3 mL of peripheral blood was collected in vials containing ethylenediamine tetraacetate acid from every participant, and DNA was extracted using a Blood Genomic DNA Extraction Kit (Xiamen Kaiso Biotech Co., Ltd., RC1001). The extracted DNA was assessed for purity (absorbance ratio of 260/280 nm between 1.8 and 2.0), and concentration using a UV spectrophotometer. After genetic counseling, pregnant women who were SMA carriers underwent interventional prenatal diagnosis and 10 mL amniotic fluid that was extracted at 18–24 weeks gestation was taken for detection. Genomic DNA was extracted using a Tissue Genome DNA Extraction Kit (Tiangen Biochemical Technology Co., Ltd., DP304, Peking), and the final DNA concentration was adjusted to between 20 and 40 ng/μL.

### 2.3 Quantitative real-time PCR

The detection reagent kit is provided by Shanghai Medical Research Co., Ltd. to detect the copy numbers of *SMN1* gene E7 and E8. The total volume of each reaction is 20 μL and there were five controls for each assay, including a blank control with no DNA, a positive control for *SMN1* E7 and E8 deletions, and three *SMN1* two-copy ladder controls. The E7 and E8 PCR primers of *SMN1* were designed based on the article reported by Smith M (20). Furthermore, the human RPP40 gene was used as the internal standard to enhance the effectiveness of the detection. The PCR amplification was performed according to this condition: 10 min at 95°C followed by 40 cycles of 95°C for 15 s and 58°C for 60 s. The fluorescence data were collected from the FAM and VIC channels, and the result of qPCR was calculated using the cycle threshold (Ct) value. In the E7 and E8 reaction, ^△^Ct_c is the value of the difference between FAM and VIC channels obtained by diluting the control at different ratios (1:2:4), and ^△^Ct_s is the difference between the target gene and the internal standard gene in the E7 and E8 reactions of the sample to be tested. The ^△△^Ct is calculated as follows:


C△△t=∑i△ Ct:c3− △Ct:s,i=1,2,4

The *i* indicates the dilution of the control. ^△△^Ct > 0.8 or is no amplification signal, and ^△△^Ct > 1.5 or a lack of amplification signal signifies the presence of a homozygous deletion in both Exon7 and Exon8 regions of the *SMN1* gene. If −0.45 < ^△△^Ct ≤ 0.45 indicated a heterozygous deletion in both Exon7 and Exon8 regions of the *SMN1* gene and ^△△^Ct ≤ −0.55 indicated normal of Exon7 and Exon8 of *SMN1* gene.

### 2.4 Multiplex Ligation-dependent Probe Amplification (MLPA) assay

For the authenticity of the results, we verified the positive and critical results of qPCR using MLPA P060 kit. SALSA MLPA Kit (P060) produced by MRC-company of the Netherlands was used, which contains 21 probes with amplification products including two probes each for *SMN1* and *SMN2* and 17 reference probes. We strictly followed the standard procedure of the SMA MLPA kit to perform experiments. DNA samples were diluted to approximately equal concentrations (20–40 ng/μL). After subjecting the samples to denaturation, hybridization, ligation, and amplification, the resulting products were analyzed utilizing the ABI 3500 genetic analyzer from Thermo Fisher Scientific. The initial data obtained were then processed and analyzed using the Coffalyser.net.

### 2.5 Statistical analyses

We performed statistical analysis using R (V4.2.1). The data was presented in the form of percentages and their 95% confidence intervals were calculated. All values were retained to two decimal places. Statistical significance was indicated by a *p* value of less than 0.05. To compare the prevalence of SMA carriers among various regions, we looked at previously published studies conducted in China within the past few years.

## 3 Results

### 3.1 The result of screening

A total of 39,647 blood samples of women were successfully tested, including 7,231 pre-pregnancy participants and 32,416 pregnancy participants. Among the 39,647 participants performed by qPCR, 726 asymptomatic SMA carriers were identified, including 134 pre-pregnancy carriers and 592 pregnancy carriers, with a carrier rate of 1.83% (95% confidence interval: 0.86–2.8%). After analysis, among the 726 asymptomatic carriers, 676 carried a single copy of both E7 and E8 and 50 carried a single copy of E7 only.

### 3.2 MLPA further confirms the results of qPCR

When using qPCR for SMA carrier screening, it is necessary to further validate the positive results of qPCR by MLPA methods (21). Therefore, we simultaneously performed MLPA testing on these 726 asymptomatic carriers. After MLPA validation, 726 asymptomatic SMA carriers were confirmed, while some variants in *SMN2* E7 and E8 were detected (Supplementary Figure S3). There were 41 cases of *SMN1* duplication, including 28 E7 single copy and 13 E8 single and double copy, and 157 cases of *SMN2* duplication, including 23 only E7 duplication, 83 E7 and E8 duplication, and only 51 E8 duplication (Supplementary Figure S4; Supplementary Table S1).

### 3.3 Prenatal diagnosis

After undergoing screening, a total of 592 pregnancy carriers were provided with genetic counseling, which included information on the etiology, genetic pattern, clinical characteristics, reproductive risk, and treatment options for SMA. Out of these carriers, 503 of their spouses (84.97, 95% confidence interval: 82.09–87.85%) voluntarily underwent SMA screening, which resulted in the identification of thirteen couples who were both SMA carriers. As a result, amniocentesis was performed on the high-risk fetuses of these thirteen couples. The findings revealed that two fetuses had a homozygous deletion of E7 and E8 of *SMN1* and two fetuses with compound heterozygosity for E7 homozygous deletion and E8 heterozygous deletion of *SMN1*, and six fetuses were carriers (Table 3). After receiving adequate genetic counseling and according to the wishes of pregnant women and couples, the parents of those high-risk fetus decided to terminate the pregnancy. On the other hand, the remaining six fetuses had a heterozygous deletion of E7 and E8 of *SMN1*, which indicated that they were SMA carriers. Despite this, the parents of these fetuses chose to continue their pregnancy.

**Table 3:** Results of the 32,416 pregnant women SMA carrier screening.

Screening date	2020.03–2020.12	2021.01–2021.06	2021.07–2021.12	2022.01–2022.06	2022.07–2022.12	2023.01–2022.04
Screened women (*n*)	4,200	4,474	6,658	5,657	6,352	5,075
Number of carriers	83	71	109	114	113	102
Carrier rate (%)	1.98	1.59	1.64	2.02	1.78	2.01
95% CI (%)	1.56–2.40	1.22–1.95	1.33–1.94	1.65–2.38	1.45–2.10	1.62–2.40
Spouse (*n*)	51	53	98	105	108	88
Recall rate (%)	61.44	74.64	89.90	92.11	95.57	86.27
95% CI (%)	50.97–71.91	64.52–84.76	84.24–95.56	87.16–97.06	91.78–99.36	79.59–92.95
Number of carrier couples	2	2	2	3	2	2
Prenatal diagnoses (*n*)	2	2	2	3	2	2
Carriers (*n*)	2	0	1	1	1	1
Affected cases (*n*)	0	1	0	1	1	1
Pregnancies terminated (*n*)	0	1	0	1	1	1

## 4 Discussion

SMA, a serious and high-incidence autosomal recessive neuromuscular disease, has a wide variability in age of onset and may develop in any ethnic group and age from birth to adulthood (22). The main manifestation is progressive muscle weakness mainly in the proximal extremities, and as the disease progresses, multi-system involvement such as respiratory, digestive, and skeletal systems may occur. Fortunately, there were many ways to screen for SMA to prevent the birth of a child with SMA (23–25). If both spouses are SMA carriers, their offspring have a 25% probability of having SMA or the normal genotype, and a 50% probability of being carriers. Therefore, preconception screening is the only reliable, economical, and safe way to prevent SMA in offspring.

Recently, there has been a growing interest in SMA carrier screening, particularly in populations with a higher disease prevalence. The availability of new genetic testing technologies has made it easier to identify carriers of the *SMN1* gene mutation, which can help inform reproductive decisions and improve patient outcomes. In recent decades, pre-pregnancy and pregnant women accepted routine prenatal screening for chromosome abnormalities in China, which has effectively reduced birth defects. However, SMA carrier screening is not a routine prenatal examination program, but it may become a prevalent screening program in the future due to its high carrier rate and severe clinical phenotype as well as the increasing acceptance of the program by pregnant women. It is important to note that both ACOG and ACMG recommend that carrier screening for SMA should be offered to all women who are planning on becoming pregnant or who are currently pregnant. This testing can help identify carriers of the SMA gene and inform couples about their risk of having a child with the condition. Ultimately, carrier screening can help individuals make informed reproductive decisions and take appropriate steps to ensure the health of their future children (10, 26).

As of the current, SMA carriers screening is already open worldwide (14, 27–29), and even has been conducted in most areas of China (16, 17, 19, 30–34). In this study, we identified 726 SMA carriers from 39,647 participants who were screened for SMA in Lanzhou City, Gansu province. The carrier rate approximately was 1/55 (1.83%), which is higher than other regions of China (Table 4), such as Liuzhou, Zhaoqing, Guangdong, Guangxi, Hainan, Yunnan. Meanwhile, the SMA carrier rate of our report was consistent with the frequency of 1/40–1/60 in the literature (20). Nevertheless, there was no significant variation in the carrier rate of SMA among different regions within China. One study that investigated 10,585 healthy couples from 34 ethnic groups in southern China with an NGS-based method, showed that there were no significant differences in SMA carrier rate among provinces, but there were differences in carrier rate among ethnic groups (16). In addition, the results of another screening study with a large pan-ethnic population showed a detection rate of 91.2% for SMA, and a carrier rate of SMA in the range of 1/47–1/68, which had no significant differences in those carrier rates (3). These studies indicate that there may be no geographical or racial differences in SMA carrier rate. In this study, race could not be summarized due to a lack of the relevant information of participants. However, our screening participants all belonged to the same area, so there was no geographic variability.

**Table 4:** SMA carrier rate in the different areas of China and different countries.

Areas	Number of screening (*n*)	SMA carriers (*n*)	Carrier frequency (%)	References
Taiwan	107,611	2,262	1/48 (2.10)	(30)
Shanghai	4,719	90	1/55 (1.90)	(31)
Sichuan	427	9	1/47 (2.11)	(35)
Liuzhou	4,931	61	1/80 (1.20)	(32)
Yunnan	3,049	62	1/49 (2.03)	(33)
Hongkong	569	9	1/63 (1.60)	(36)
Zhaoqing	5,200	75	1/69 (1.44)	(19)
Warsaw	1,076	26	1/41 (2.44)	(37)
Poland	600	17	1/35 (2.86)	(38)
Saudi	4,090	108	1/38 (2.63)	(14)
Indian	606	16	1/38 (2.63)	(15)
Thailand	505	9	1/56 (1.78)	(27)
Guangdong	4,755	60	1/79 (1.30)	(16)
Guangxi	5,009	71	1/71 (1.40)	(16)
Hainan	2,778	45	1/62 (1.60)	(16)
Yunnan	4,049	66	1/61 (1.60)	(16)
Guizhou	4,292	41	1/104 (1.00)	(16)
Gansu	39,647*	726	1/55 (1.83)	This study

^*^Includes 32,416 pregnant women and 7,231 pre-pregnant women.

Since *SMN1* and *SMN2* exhibit such a high level of sequence identity, previous research efforts have not fully leveraged the vantage of Next Generation Sequencing (NGS) technology. SMA carrier screening in domestic areas of China is now basically based on RT-PCR testing of specific regions of the *SMN1* gene, which cannot comprehensively cover the *SMN1* gene, especially single-nucleotide variants (SNVs) and insertions/deletions (Indels). The recent development of NGS-based technology for SMA carrier screening is gradually being used in the clinical, making SMA testing part of a comprehensive NGS carrier testing platform. Previous studies compared and validated the performances of the sequencing based and MLPA carrier status detection technologies, which shows a strong association between them (39). NGS-based screening technology for SMA carriers is expected to provide comprehensive coverage of the *SMN1* and *SMN2* genes and more scientific and detailed explanations of the causes of different clinical phenotypes of SMA. The different clinical phenotypes of SMA were further explained by increasing the depth of sequencing and optimizing the algorithms of data analysis to accurately call copy number variants of *SMN1* and *SMN2*. In this study, SMA carrier screening was conducted in three stages. First, we used RT-PCR to identify the copy number of E7 and E8 of the *SMN1* and *SMN2* genes, and we used MLPA to validate the abnormal results obtained by RT-PCR in pregnant women. Second, if the pregnant woman was identified as a carrier, her spouse should be recommended to accept SMA carrier screening. Finally, we proposed genetic counseling and prenatal diagnosis if the couples were confirmed as carriers. It is important that prenatal counseling for individuals or couples planning to conceive should include information related to Spinal Muscular Atrophy (SMA). This should include guidance on the genetic causes and transmission mode of SMA, recurrence risk assessment, options for prenatal diagnosis or preimplantation genetic testing, and recommendations for carrier screening of family members. By providing this information, families can make informed decisions about their reproductive choices and better understand the potential risks and implications associated with SMA. Healthcare providers must work together with patients and their families to provide comprehensive prenatal counseling and support (13).

In this study, out of the 726 prepregnant women and pregnant women who were identified as carriers during screening, only the spouses of 623 of them were advised to undergo SMA screening. The recall rate for this advice was 85.81% (95% confidence interval: 83.27–88.35%). Except for the families who were not yet ready for screening due to low gestational weeks, about 30% of spouses who were not recalled may be due to poor economic conditions or less recognition of SMA. At the same time, we also analyzed the SMA carrier status of these spouses, and the result showed a carrier rate of 2.08% (95% confidence interval: 0.96–3.2%), which is higher than the combined carrier rate in many countries. This may be because some spouses were not recalled. After undergoing thorough genetic counseling, the couple was advised to undergo prenatal diagnosis for their fetuses. Among the three fetuses identified as being at high risk for SMA, two were found to carry a heterozygous deletion of *SMN1* E7 and E8 genes, while one had a homozygous deletion of the same genes, which would have resulted in SMA after birth. After additional counseling, the parents of the latter opted for pregnancy termination. For the 28 women who were SMA carriers but did not have their partners undergo screening, postnatal follow-up was provided. The follow-up results showed that some mothers chose to have their babies diagnosed with SMA after birth, while others were unwilling to be screened for SMA carriers. The study discovered that almost 30% of pregnant women’s spouses did not undergo SMA carrier screening, indicating that some newborns may have missed an early diagnosis. With SMA treatment drugs now covered by medical insurance, early detection, and diagnosis through newborn screening and other methods are crucial for effective SMA treatment.

Overall, it is crucial to recognize the significance of SMA carrier screening as a means of identifying those who may be carriers of this disease and ensuring that they receive appropriate counseling and care. The alarming rate of carriers discovered in recent studies underscores the importance of raising awareness and increasing access to screening services. Additionally, continued research into the underlying genetic mechanisms and treatment options for SMA holds great promise for improving the lives of those affected by this condition and their loved ones. However, there are some limitations in this study, we only detected copy number variants in exon 7 and exon 8 of *SMN1* and SMN2, but SNVs or Indels of *SMN1* and *SMN2* or other special types such as the 2 + 0 type are outside the scope of this study, which may have missed this group of carriers. With other innovations in technology, it may be possible to compensate for the limitations of this technique in the future. In summary, it is our collective responsibility to prioritize and support efforts aimed at combating SMA and other genetic disorders through comprehensive prevention, identification, and treatment strategies.

## Data availability statement

The raw data supporting the conclusions of this article will be made available by the authors, without undue reservation.

## Ethics statement

The studies involving humans were approved by Institutional Review Boards of Gansu Provincial Maternity and Child-care Hospital. The studies were conducted in accordance with the local legislation and institutional requirements. Written informed consent for participation in this study was provided by the participants’ legal guardians/next of kin.

## Author contributions

B-bZ: Data curation, Visualization, Writing – original draft. XC: Methodology, Writing – review & editing. CZ: Investigation, Writing – review & editing. Y-pW: Validation, Writing – review & editing. P-pM: Resources, Writing – review & editing. S-jH: Conceptualization, Writing – review & editing. LH: Funding acquisition, Writing – review & editing. Y-fB: Supervision, Writing – review & editing.
