# Supplementary material for: Analysis of spinal muscular atrophy carrier screening results in 32,416 pregnant women and 7,231 prepregnant women
Source: Front Neurol. 2024 Apr 9;15:1357476. doi: 10.3389/fneur.2024.1357476 (PMC11035774; doi:10.3389/fneur.2024.1357476)
Supplement: Supplementary file 1 [file Data_Sheet_1.pdf]

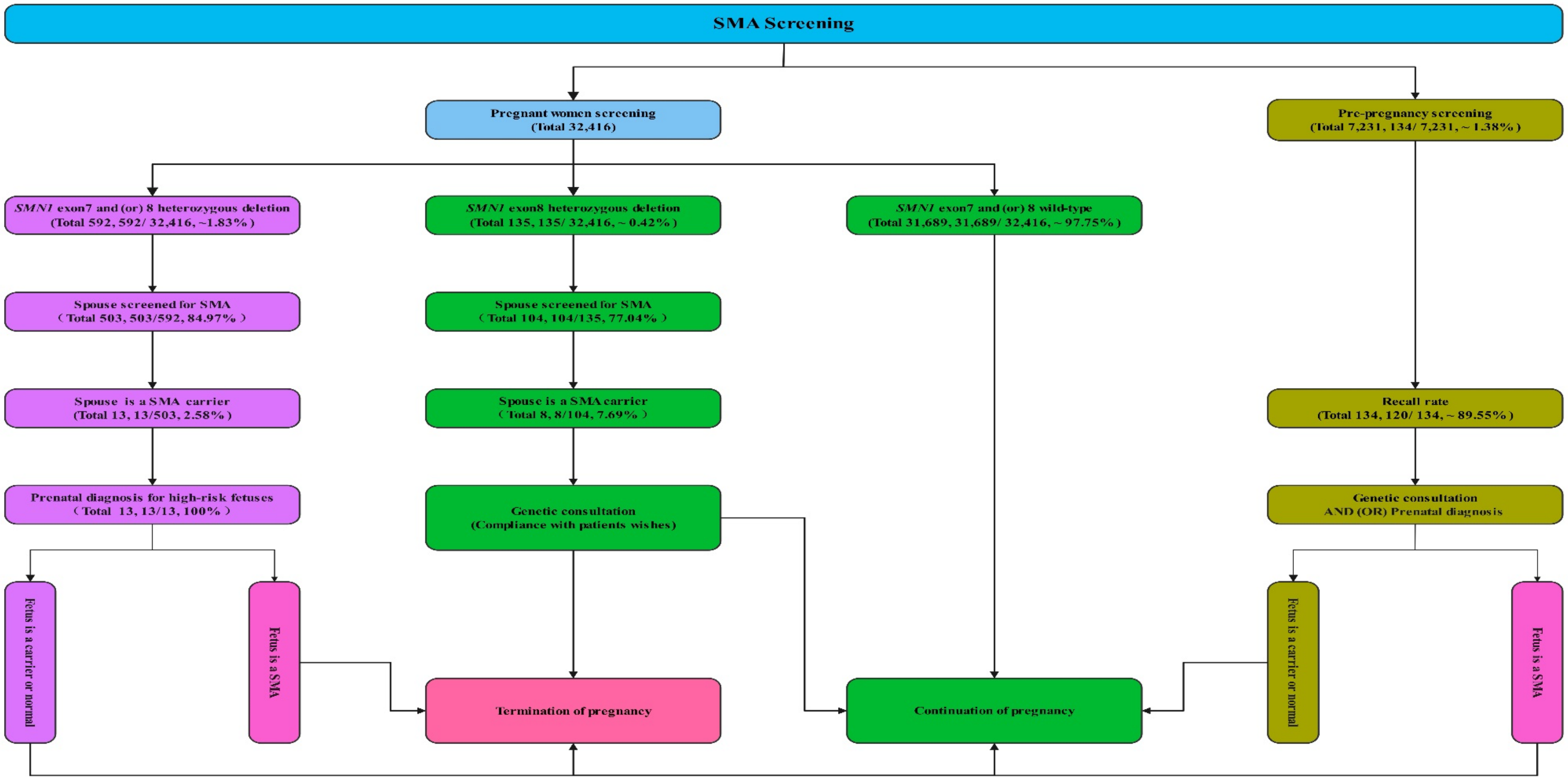

Figure S1 The detailed flow chart of this study. The different colors represent distinct clinical pathways in the figure, where purple is the first clinical diagnostic pathway used when the participant was a heterozygous deletion of E7 and/or E8 of *SMN1*, and green is the second clinical diagnostic pathway used when the participant was a wild-type or E8 heterozygous deletion of *SMN1*. The brown color represents the clinical pathway for pre-pregnant women.

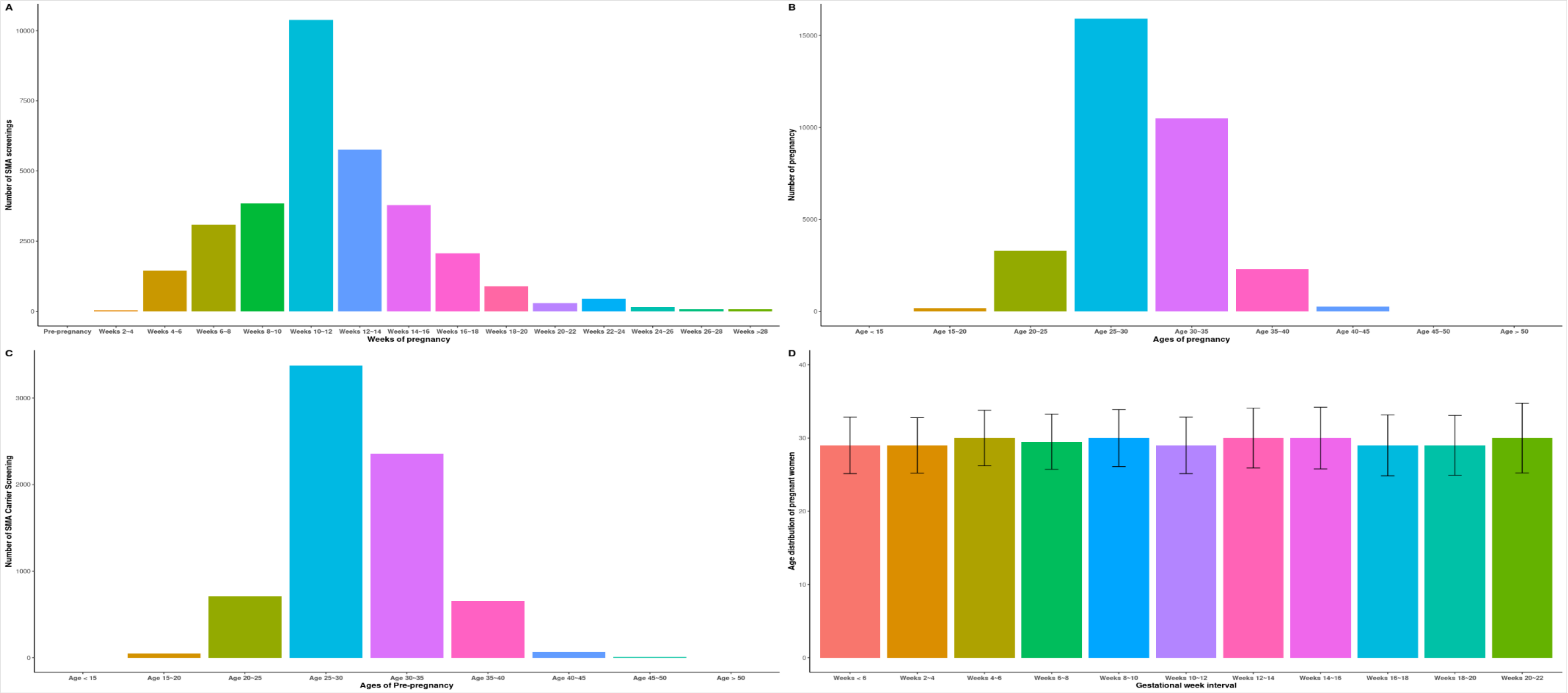

Figure S2 The statistical analysis of the age and gestational week of pre-pregnancy and pregnancy women. A shows that the distribution of the number of participants at different stages of pregnancy. B and C show that the age distribution of pregnant women and prepregnant women, respectively. D shows that the age distribution of participants in different gestational week intervals, and there were no differences in the age of pregnant women with different gestational weeks.

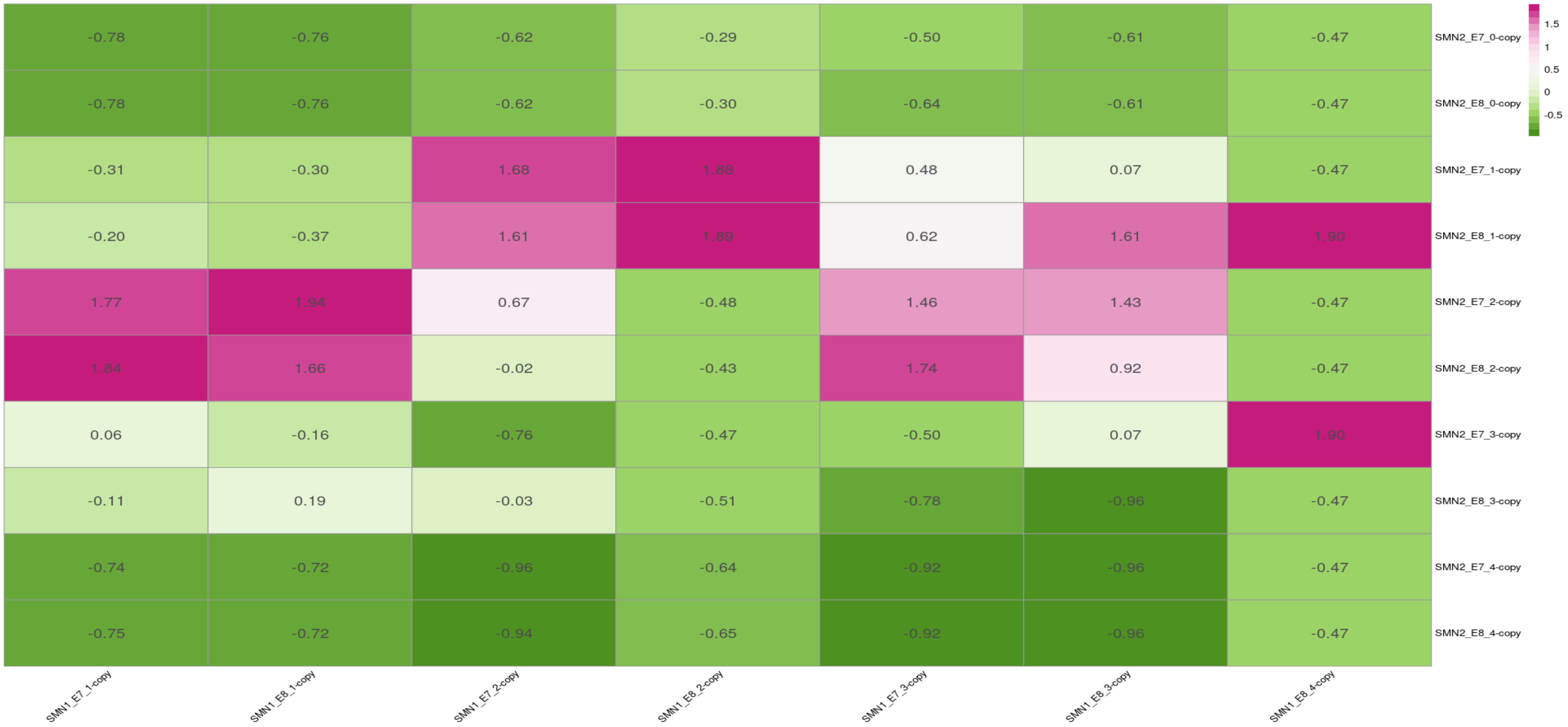

Figure S3 Genotyping analysis of spinal muscular atrophy via qPCR and MLPA. The Fuchsia color indicates more cases, and the green color indicates fewer cases. The X-axis represents the *SMN1*, where SMN1_E*_#-copy indicates that the copy number of exon * of the *SMN1* was #. For example, SMN1_E7_1-copy indicates that exon 7 of the *SMN1* was 1 copy. The Y-axis represents the *SMN2*, where SMN2_E*_#-copy indicates that the copy number of exon * of the *SMN2* was #. For example, SMN2_E7_1-copy indicates that exon 7 of the *SMN2* was 1 copy. The values in the figure indicate the normalized values for the number of individuals for the *SMN1* and *SMN2* variants.

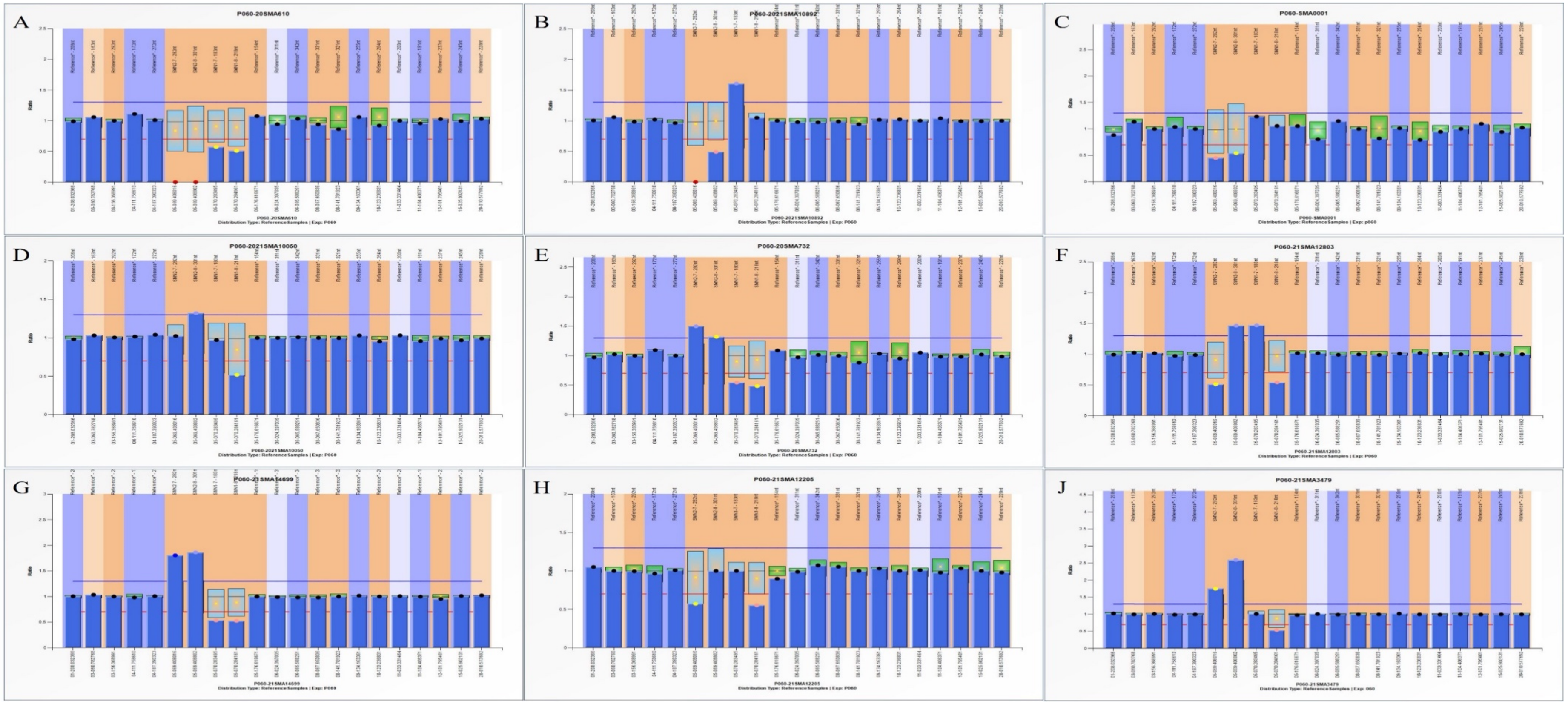

Figure S4 Presentation of selected results confirmed by MLPA methodology. *SMN2* was in the front, and *SMN1* was in the back. A shows that the E7 and E8 of *SMN2* were both 0 copies, and the E7 and E8 of *SMN1* were both 1 copy. B shows that the E7 of *SMN2* was 0 copies and the E8 of *SMN2* was 1 copy. C shows that the E7 and E8 of *SMN2* were both 1 copy. D shows that the E8 of *SMN2* was 3 copies, and the E8 of *SMN1* was 1 copy. E shows that the E7 and E8 of *SMN2* were both 3 copies, and the E7 and E8 of *SMN1* were both 1 copy. F shows that the E7 of *SMN2* and E8 of *SMN1* were both 1 copy, and the E8 of *SMN2* and E7 of *SMN1* were both 3 copies. G shows that the E7 and E8 of *SMN2* were both 4 copies, and the E7 and E8 of *SMN1* were both 1 copy. H shows that the E7 of *SMN2* and E8 of *SMN1* were both 1 copy. J shows that the E7 and E8 of *SMN2* were 3 copies and 4 copies, respectively, and the E8 of SMN1 was 1 copy.

Supplementary Table 1 The result of MLPA method validation

Year	No.	*SMN1*		*SMN2*	
		Exon7	Exon8	Exon7	Exon8
2020	1	2 copies	2 copies	1 copy	1 copy
2020	2	2 copies	2 copies	1 copy	1 copy
2020	3	1 copy	1 copy	2 copies	2 copies
2020	4	2 copies	2 copies	1 copy	1 copy
2020	5	1 copy	1 copy	2 copies	2 copies
2020	6	2 copies	1 copy	2 copies	2 copies
2020	7	1 copy	1 copy	2 copies	2 copies
2020	8	1 copy	1 copy	0 copy	0 copy
2020	9	1 copy	1 copy	3 copies	3 copies
2020	10	1 copy	1 copy	2 copies	2 copies
2020	11	1 copy	1 copy	1 copy	1 copy
2020	12	1 copy	1 copy	2 copies	2 copies
2020	13	1 copy	1 copy	4 copies	2 copies
2020	14	1 copy	1 copy	2 copies	2 copies
2020	15	1 copy	1 copy	2 copies	2 copies
2020	16	1 copy	1 copy	2 copies	2 copies
2020	17	2 copies	1 copy	2 copies	2 copies
2020	18	1 copy	2 copies	2 copies	2 copies
2020	19	1 copy	2 copies	2 copies	2 copies
2020	20	2 copies	2 copies	1 copy	1 copy
2020	21	1 copy	1 copy	2 copies	2 copies

2020	22	1 copy	2 copies	2 copies	2 copies
2020	23	2 copies	1 copy	2 copies	2 copies
2020	24	2 copies	1 copy	2 copies	2 copies
2020	25	2 copies	1 copy	2 copies	2 copies
2020	26	1 copy	1 copy	2 copies	2 copies
2020	27	1 copy	1 copy	2 copies	2 copies
2020	28	1 copy	1 copy	2 copies	2 copies
2020	29	1 copy	1 copy	2 copies	2 copies
2020	30	1 copy	1 copy	2 copies	2 copies
2020	31	1 copy	1 copy	2 copies	2 copies
2020	32	2 copies	1 copy	2 copies	2 copies
2020	33	1 copy	1 copy	2 copies	2 copies
2020	34	1 copy	1 copy	2 copies	2 copies
2020	35	1 copy	1 copy	2 copies	2 copies
2020	36	2 copies	1 copy	2 copies	2 copies
2020	37	1 copy	1 copy	2 copies	2 copies
2020	38	1 copy	1 copy	2 copies	2 copies
2020	39	1 copy	2 copies	2 copies	2 copies
2020	40	1 copy	1 copy	2 copies	2 copies
2020	41	2 copies	1 copy	2 copies	2 copies
2020	42	1 copy	1 copy	2 copies	2 copies
2020	43	1 copy	2 copies	2 copies	2 copies
2020	44	1 copy	1 copy	2 copies	2 copies
2020	45	1 copy	1 copy	2 copies	2 copies
2020	46	1 copy	1 copy	2 copies	2 copies

2020	47	1 copy	1 copy	2 copies	2 copies
2020	48	1 copy	1 copy	2 copies	2 copies
2020	49	1 copy	1 copy	2 copies	2 copies
2020	50	1 copy	1 copy	2 copies	2 copies
2020	51	1 copy	1 copy	2 copies	2 copies
2020	52	2 copies	1 copy	2 copies	2 copies
2020	53	1 copy	1 copy	2 copies	2 copies
2020	54	2 copies	1 copy	2 copies	2 copies
2020	55	1 copy	1 copy	2 copies	2 copies
2020	56	2 copies	1 copy	2 copies	2 copies
2020	57	1 copy	1 copy	2 copies	2 copies
2020	58	2 copies	1 copy	2 copies	2 copies
2020	59	2 copies	1 copy	2 copies	2 copies
2020	60	2 copies	1 copy	2 copies	2 copies
2020	61	2 copies	1 copy	2 copies	2 copies
2020	62	2 copies	1 copy	2 copies	2 copies
2020	63	1 copy	1 copy	2 copies	2 copies
2020	64	2 copies	1 copy	2 copies	2 copies
2020	65	3 copies	3 copies	2 copies	1 copy
2020	66	1 copy	1 copy	2 copies	2 copies
2020	67	1 copy	1 copy	2 copies	2 copies
2020	68	2 copies	1 copy	2 copies	2 copies
2020	69	1 copy	1 copy	2 copies	2 copies
2020	70	2 copies	1 copy	2 copies	2 copies
2020	71	2 copies	1 copy	2 copies	2 copies

2020	72	1 copy	1 copy	2 copies	2 copies
2020	73	2 copies	1 copy	2 copies	2 copies
2020	74	1 copy	1 copy	2 copies	2 copies
2020	75	2 copies	1 copy	2 copies	2 copies
2020	76	1 copy	1 copy	2 copies	2 copies
2020	77	2 copies	1 copy	2 copies	2 copies
2020	78	1 copy	1 copy	2 copies	2 copies
2020	79	1 copy	1 copy	2 copies	2 copies
2020	80	1 copy	1 copy	2 copies	2 copies
2020	81	1 copy	1 copy	2 copies	2 copies
2020	82	1 copy	1 copy	2 copies	2 copies
2020	83	1 copy	1 copy	2 copies	2 copies
2020	84	1 copy	1 copy	2 copies	2 copies
2020	85	1 copy	1 copy	2 copies	2 copies
2020	86	1 copy	1 copy	2 copies	2 copies
2020	87	1 copy	1 copy	2 copies	2 copies
2020	88	2 copies	1 copy	2 copies	2 copies
2020	89	1 copy	1 copy	2 copies	2 copies
2020	90	1 copy	1 copy	2 copies	2 copies
2020	91	2 copies	1 copy	2 copies	2 copies
2020	92	1 copy	1 copy	2 copies	2 copies
2020	93	1 copy	2 copies	2 copies	2 copies
2020	94	1 copy	1 copy	2 copies	2 copies
2020	95	1 copy	2 copies	2 copies	2 copies
2020	96	1 copy	1 copy	2 copies	2 copies

2020	97	1 copy	1 copy	2 copies	2 copies
2020	98	1 copy	1 copy	2 copies	2 copies
2020	99	1 copy	1 copy	1 copy	1 copy
2020	100	1 copy	1 copy	2 copies	2 copies
2020	101	2 copies	2 copies	1 copy	1 copy
2020	102	1 copy	2 copies	2 copies	2 copies
2020	103	1 copy	2 copies	2 copies	2 copies
2020	104	1 copy	1 copy	2 copies	2 copies
2021	105	2 copies	1 copy	2 copies	2 copies
2021	106	2 copies	1 copy	2 copies	2 copies
2021	107	1 copy	1 copy	2 copies	2 copies
2021	108	1 copy	1 copy	2 copies	2 copies
2021	109	1 copy	1 copy	2 copies	2 copies
2021	110	1 copy	1 copy	2 copies	2 copies
2021	111	1 copy	2 copies	2 copies	2 copies
2021	112	1 copy	1 copy	2 copies	2 copies
2021	113	1 copy	1 copy	2 copies	2 copies
2021	114	1 copy	1 copy	2 copies	2 copies
2021	115	2 copies	1 copy	2 copies	2 copies
2021	116	1 copy	1 copy	2 copies	2 copies
2021	117	1 copy	1 copy	2 copies	2 copies
2021	118	2 copies	1 copy	2 copies	2 copies
2021	119	1 copy	1 copy	2 copies	2 copies
2021	120	1 copy	1 copy	2 copies	2 copies
2021	121	1 copy	1 copy	2 copies	2 copies

2021	122	1 copy	1 copy	2 copies	2 copies
2021	123	1 copy	1 copy	2 copies	2 copies
2021	124	1 copy	1 copy	2 copies	2 copies
2021	125	1 copy	1 copy	2 copies	2 copies
2021	126	1 copy	1 copy	2 copies	2 copies
2021	127	1 copy	1 copy	2 copies	2 copies
2021	128	2 copies	1 copy	2 copies	2 copies
2021	129	1 copy	1 copy	2 copies	2 copies
2021	130	2 copies	1 copy	2 copies	3 copies
2021	131	1 copy	2 copies	2 copies	1 copy
2021	132	2 copies	1 copy	2 copies	3 copies
2021	133	2 copies	1 copy	2 copies	3 copies
2021	134	1 copy	1 copy	2 copies	2 copies
2021	135	2 copies	2 copies	1 copy	1 copy
2021	136	1 copy	1 copy	1 copy	1 copy
2021	137	1 copy	1 copy	2 copies	2 copies
2021	138	2 copies	1 copy	2 copies	3 copies
2021	139	2 copies	1 copy	2 copies	3 copies
2021	140	1 copy	1 copy	2 copies	2 copies
2021	141	1 copy	1 copy	1 copy	1 copy
2021	142	1 copy	1 copy	2 copies	2 copies
2021	143	3 copies	2 copies	0 copy	1 copy
2021	144	1 copy	1 copy	3 copies	3 copies
2021	145	1 copy	1 copy	2 copies	2 copies
2021	146	2 copies	1 copy	2 copies	3 copies

2021	147	1 copy	1 copy	2 copies	2 copies
2021	148	1 copy	1 copy	3 copies	3 copies
2021	149	1 copy	1 copy	2 copies	2 copies
2021	150	2 copies	1 copy	2 copies	3 copies
2021	151	1 copy	1 copy	3 copies	3 copies
2021	152	1 copy	1 copy	2 copies	2 copies
2021	153	1 copy	1 copy	1 copy	1 copy
2021	154	3 copies	3 copies	2 copies	2 copies
2021	155	1 copy	1 copy	2 copies	2 copies
2021	156	1 copy	1 copy	1 copy	1 copy
2021	157	1 copy	1 copy	3 copies	3 copies
2021	158	1 copy	1 copy	2 copies	2 copies
2021	159	1 copy	2 copies	3 copies	2 copies
2021	160	2 copies	1 copy	2 copies	3 copies
2021	161	1 copy	1 copy	2 copies	2 copies
2021	162	2 copies	1 copy	1 copy	2 copies
2021	163	1 copy	1 copy	3 copies	3 copies
2021	164	1 copy	1 copy	2 copies	2 copies
2021	165	1 copy	1 copy	2 copies	2 copies
2021	166	1 copy	1 copy	3 copies	3 copies
2021	167	2 copies	1 copy	2 copies	3 copies
2021	168	2 copies	1 copy	2 copies	3 copies
2021	169	1 copy	1 copy	2 copies	2 copies
2021	170	2 copies	1 copy	2 copies	3 copies
2021	171	2 copies	3 copies	2 copies	2 copies

2021	172	1 copy	1 copy	2 copies	2 copies
2021	173	1 copy	1 copy	2 copies	2 copies
2021	174	2 copies	1 copy	2 copies	3 copies
2021	175	1 copy	1 copy	1 copy	1 copy
2021	176	1 copy	1 copy	2 copies	2 copies
2021	177	3 copies	1 copy	1 copy	3 copies
2021	178	1 copy	1 copy	2 copies	2 copies
2021	179	1 copy	1 copy	3 copies	3 copies
2021	180	3 copies	2 copies	1 copy	2 copies
2021	181	1 copy	2 copies	3 copies	2 copies
2021	182	1 copy	2 copies	3 copies	2 copies
2021	183	1 copy	1 copy	1 copy	1 copy
2021	184	2 copies	1 copy	2 copies	3 copies
2021	185	1 copy	1 copy	3 copies	3 copies
2021	186	2 copies	1 copy	2 copies	3 copies
2021	187	2 copies	1 copy	2 copies	2 copies
2021	188	3 copies	3 copies	0 copy	0 copy
2021	189	2 copies	3 copies	2 copies	1 copy
2021	190	1 copy	1 copy	2 copies	2 copies
2021	191	1 copy	1 copy	2 copies	2 copies
2021	192	1 copy	1 copy	1 copy	1 copy
2021	193	2 copies	1 copy	2 copies	3 copies
2021	194	1 copy	1 copy	2 copies	2 copies
2021	195	3 copies	3 copies	1 copy	1 copy
2021	196	1 copy	1 copy	2 copies	2 copies

2021	197	1 copy	2 copies	3 copies	2 copies
2021	198	2 copies	1 copy	2 copies	1 copy
2021	199	1 copy	1 copy	2 copies	2 copies
2021	200	1 copy	1 copy	3 copies	3 copies
2021	201	1 copy	1 copy	2 copies	2 copies
2021	202	2 copies	1 copy	2 copies	3 copies
2021	203	1 copy	1 copy	3 copies	3 copies
2021	204	1 copy	1 copy	3 copies	3 copies
2021	205	1 copy	1 copy	2 copies	2 copies
2021	206	1 copy	2 copies	3 copies	2 copies
2021	207	1 copy	1 copy	2 copies	2 copies
2021	208	1 copy	1 copy	2 copies	2 copies
2021	209	1 copy	1 copy	1 copy	1 copy
2021	210	1 copy	1 copy	2 copies	2 copies
2021	211	1 copy	1 copy	2 copies	2 copies
2021	212	2 copies	1 copy	2 copies	3 copies
2021	213	1 copy	1 copy	3 copies	3 copies
2021	214	1 copy	1 copy	2 copies	2 copies
2021	215	1 copy	1 copy	3 copies	3 copies
2021	216	1 copy	1 copy	3 copies	3 copies
2021	217	1 copy	1 copy	1 copy	1 copy
2021	218	1 copy	1 copy	2 copies	2 copies
2021	219	1 copy	1 copy	1 copy	1 copy
2021	220	1 copy	1 copy	2 copies	2 copies
2021	221	1 copy	1 copy	3 copies	3 copies

2021	222	1 copy	1 copy	4 copies	4 copies
2021	223	1 copy	1 copy	2 copies	2 copies
2021	224	1 copy	1 copy	2 copies	2 copies
2021	225	1 copy	1 copy	3 copies	3 copies
2021	226	1 copy	1 copy	3 copies	3 copies
2021	227	1 copy	2 copies	2 copies	1 copy
2021	228	1 copy	1 copy	2 copies	2 copies
2021	229	1 copy	1 copy	2 copies	2 copies
2021	230	1 copy	1 copy	2 copies	2 copies
2021	231	2 copies	1 copy	2 copies	2 copies
2021	232	1 copy	1 copy	2 copies	2 copies
2021	233	1 copy	1 copy	2 copies	2 copies
2021	234	1 copy	1 copy	2 copies	2 copies
2021	235	2 copies	1 copy	2 copies	2 copies
2021	236	1 copy	1 copy	2 copies	2 copies
2021	237	1 copy	1 copy	2 copies	2 copies
2021	238	1 copy	1 copy	2 copies	2 copies
2021	239	1 copy	1 copy	3 copies	3 copies
2021	240	1 copy	1 copy	2 copies	2 copies
2021	241	1 copy	1 copy	2 copies	2 copies
2021	242	1 copy	1 copy	4 copies	4 copies
2021	243	1 copy	1 copy	2 copies	2 copies
2021	244	1 copy	1 copy	2 copies	2 copies
2021	245	1 copy	1 copy	2 copies	2 copies
2021	246	2 copies	1 copy	2 copies	2 copies

2021	247	1 copy	1 copy	2 copies	2 copies
2021	248	1 copy	1 copy	2 copies	2 copies
2021	249	1 copy	1 copy	3 copies	3 copies
2021	250	2 copies	1 copy	2 copies	2 copies
2021	251	1 copy	1 copy	3 copies	3 copies
2021	252	1 copy	1 copy	2 copies	2 copies
2021	253	1 copy	1 copy	2 copies	2 copies
2021	254	2 copies	1 copy	2 copies	3 copies
2021	255	1 copy	1 copy	3 copies	2 copies
2021	256	1 copy	1 copy	2 copies	2 copies
2021	257	1 copy	1 copy	2 copies	2 copies
2021	258	2 copies	1 copy	2 copies	3 copies
2021	259	1 copy	1 copy	3 copies	3 copies
2021	260	2 copies	1 copy	2 copies	2 copies
2021	261	1 copy	1 copy	2 copies	2 copies
2021	262	1 copy	1 copy	2 copies	2 copies
2021	263	1 copy	1 copy	2 copies	2 copies
2021	264	1 copy	3 copies	3 copies	1 copy
2021	265	1 copy	2 copies	2 copies	2 copies
2021	266	1 copy	1 copy	2 copies	2 copies
2021	267	2 copies	1 copy	2 copies	2 copies
2021	268	1 copy	1 copy	2 copies	2 copies
2021	269	1 copy	1 copy	3 copies	3 copies
2021	270	1 copy	1 copy	3 copies	3 copies
2021	271	2 copies	1 copy	2 copies	2 copies

2021	272	2 copies	1 copy	3 copies	4 copies
2021	273	1 copy	1 copy	3 copies	3 copies
2021	274	1 copy	1 copy	2 copies	2 copies
2021	275	1 copy	1 copy	2 copies	2 copies
2021	276	1 copy	1 copy	2 copies	2 copies
2021	277	2 copies	1 copy	2 copies	2 copies
2021	278	1 copy	3 copies	3 copies	1 copy
2021	279	1 copy	1 copy	1 copy	1 copy
2021	280	1 copy	1 copy	1 copy	1 copy
2021	281	1 copy	1 copy	1 copy	1 copy
2021	282	1 copy	1 copy	2 copies	2 copies
2021	283	3 copies	3 copies	1 copy	1 copy
2021	284	2 copies	1 copy	2 copies	3 copies
2021	285	1 copy	1 copy	2 copies	2 copies
2021	286	1 copy	1 copy	2 copies	2 copies
2021	287	2 copies	1 copy	1 copy	2 copies
2021	288	1 copy	1 copy	1 copy	1 copy
2021	289	1 copy	1 copy	2 copies	2 copies
2021	290	1 copy	1 copy	2 copies	2 copies
2021	291	1 copy	1 copy	2 copies	2 copies
2021	292	1 copy	2 copies	3 copies	2 copies
2021	293	1 copy	1 copy	2 copies	2 copies
2021	294	1 copy	1 copy	2 copies	2 copies
2021	295	1 copy	2 copies	3 copies	2 copies
2021	296	1 copy	1 copy	3 copies	3 copies

2021	297	1 copy	1 copy	3 copies	3 copies
2021	298	2 copies	1 copy	2 copies	2 copies
2021	299	1 copy	1 copy	3 copies	3 copies
2021	300	1 copy	1 copy	2 copies	2 copies
2021	301	2 copies	1 copy	3 copies	4 copies
2021	302	1 copy	1 copy	2 copies	2 copies
2021	303	2 copies	1 copy	2 copies	3 copies
2021	304	1 copy	1 copy	2 copies	2 copies
2021	305	1 copy	1 copy	0 copy	0 copy
2021	306	2 copies	1 copy	2 copies	3 copies
2021	307	1 copy	1 copy	2 copies	2 copies
2021	308	1 copy	1 copy	2 copies	2 copies
2021	309	1 copy	1 copy	2 copies	2 copies
2021	310	1 copy	1 copy	2 copies	2 copies
2021	311	2 copies	1 copy	2 copies	2 copies
2021	312	1 copy	1 copy	2 copies	2 copies
2021	313	1 copy	1 copy	2 copies	2 copies
2021	314	1 copy	1 copy	3 copies	3 copies
2021	315	2 copies	1 copy	2 copies	3 copies
2021	316	1 copy	1 copy	2 copies	2 copies
2021	317	1 copy	1 copy	2 copies	3 copies
2021	318	1 copy	1 copy	2 copies	2 copies
2021	319	2 copies	1 copy	2 copies	2 copies
2021	320	2 copies	1 copy	1 copy	2 copies
2021	321	1 copy	1 copy	2 copies	2 copies

2021	322	1 copy	1 copy	2 copies	2 copies
2021	323	1 copy	1 copy	3 copies	3 copies
2021	324	1 copy	1 copy	3 copies	3 copies
2021	325	1 copy	1 copy	2 copies	2 copies
2021	326	1 copy	1 copy	1 copy	1 copy
2021	327	1 copy	1 copy	2 copies	2 copies
2021	328	1 copy	1 copy	2 copies	2 copies
2021	329	1 copy	1 copy	2 copies	2 copies
2021	330	2 copies	1 copy	2 copies	2 copies
2021	331	1 copy	1 copy	2 copies	2 copies
2021	332	1 copy	1 copy	2 copies	2 copies
2021	333	1 copy	1 copy	2 copies	2 copies
2021	334	1 copy	1 copy	2 copies	2 copies
2021	335	1 copy	1 copy	2 copies	2 copies
2021	336	1 copy	1 copy	2 copies	2 copies
2021	337	1 copy	1 copy	2 copies	2 copies
2021	338	1 copy	1 copy	2 copies	2 copies
2021	339	1 copy	1 copy	2 copies	2 copies
2021	340	1 copy	1 copy	2 copies	2 copies
2021	341	1 copy	1 copy	2 copies	2 copies
2021	342	1 copy	1 copy	1 copy	1 copy
2021	343	1 copy	1 copy	2 copies	2 copies
2021	344	1 copy	1 copy	2 copies	2 copies
2021	345	1 copy	1 copy	1 copy	1 copy
2021	346	1 copy	1 copy	3 copies	3 copies

2021	347	1 copy	1 copy	2 copies	2 copies
2021	348	2 copies	1 copy	2 copies	2 copies
2021	349	1 copy	1 copy	2 copies	2 copies
2021	350	1 copy	1 copy	2 copies	2 copies
2021	351	1 copy	1 copy	2 copies	2 copies
2021	352	1 copy	1 copy	3 copies	3 copies
2021	353	1 copy	1 copy	2 copies	2 copies
2021	354	1 copy	2 copies	4 copies	2 copies
2021	355	1 copy	1 copy	2 copies	2 copies
2021	356	1 copy	1 copy	3 copies	3 copies
2021	357	1 copy	1 copy	2 copies	2 copies
2021	358	1 copy	1 copy	3 copies	3 copies
2021	359	1 copy	1 copy	2 copies	2 copies
2021	360	1 copy	1 copy	2 copies	2 copies
2021	361	1 copy	1 copy	1 copy	1 copy
2021	362	1 copy	1 copy	2 copies	2 copies
2021	363	1 copy	1 copy	3 copies	3 copies
2021	364	1 copy	3 copies	2 copies	2 copies
2021	365	2 copies	1 copy	2 copies	3 copies
2021	366	1 copy	1 copy	2 copies	2 copies
2021	367	3 copies	3 copies	2 copies	1 copy
2021	368	1 copy	1 copy	2 copies	2 copies
2021	369	1 copy	1 copy	3 copies	3 copies
2021	370	1 copy	1 copy	3 copies	3 copies
2021	371	1 copy	1 copy	2 copies	2 copies

2021	372	1 copy	1 copy	3 copies	3 copies
2021	373	1 copy	1 copy	1 copy	1 copy
2021	374	1 copy	1 copy	1 copy	1 copy
2021	375	2 copies	1 copy	2 copies	3 copies
2021	376	1 copy	1 copy	3 copies	3 copies
2021	377	2 copies	1 copy	2 copies	3 copies
2021	378	1 copy	1 copy	2 copies	2 copies
2021	379	1 copy	1 copy	2 copies	2 copies
2021	380	1 copy	1 copy	2 copies	2 copies
2021	381	1 copy	2 copies	1 copy	2 copies
2021	382	1 copy	1 copy	3 copies	3 copies
2021	383	1 copy	1 copy	3 copies	3 copies
2021	384	1 copy	1 copy	1 copy	1 copy
2021	385	1 copy	1 copy	2 copies	2 copies
2021	386	1 copy	1 copy	2 copies	2 copies
2021	387	2 copies	1 copy	1 copy	2 copies
2021	388	2 copies	1 copy	2 copies	3 copies
2021	389	3 copies	3 copies	1 copy	1 copy
2021	390	1 copy	1 copy	2 copies	2 copies
2021	391	2 copies	2 copies	1 copy	1 copy
2021	392	2 copies	2 copies	1 copy	1 copy
2021	393	2 copies	2 copies	1 copy	1 copy
2021	394	2 copies	2 copies	1 copy	1 copy
2021	395	2 copies	2 copies	1 copy	1 copy
2021	396	2 copies	2 copies	1 copy	1 copy

2021	397	2 copies	2 copies	1 copy	1 copy
2021	398	2 copies	2 copies	1 copy	1 copy
2021	399	2 copies	2 copies	3 copies	3 copies
2021	400	2 copies	2 copies	1 copy	1 copy
2021	401	2 copies	2 copies	1 copy	1 copy
2021	402	2 copies	2 copies	1 copy	1 copy
2021	403	2 copies	2 copies	1 copy	1 copy
2021	404	2 copies	2 copies	1 copy	1 copy
2021	405	2 copies	2 copies	1 copy	1 copy
2021	406	2 copies	2 copies	1 copy	1 copy
2021	407	2 copies	2 copies	1 copy	1 copy
2021	408	2 copies	2 copies	1 copy	1 copy
2021	409	2 copies	2 copies	1 copy	1 copy
2021	410	2 copies	2 copies	1 copy	1 copy
2021	411	2 copies	2 copies	1 copy	1 copy
2021	412	2 copies	2 copies	1 copy	1 copy
2021	413	2 copies	2 copies	1 copy	1 copy
2021	414	2 copies	2 copies	1 copy	1 copy
2021	415	2 copies	2 copies	1 copy	1 copy
2021	416	2 copies	2 copies	1 copy	1 copy
2021	417	2 copies	2 copies	1 copy	1 copy
2021	418	2 copies	2 copies	1 copy	1 copy
2021	419	2 copies	2 copies	1 copy	1 copy
2021	420	2 copies	2 copies	1 copy	1 copy
2021	421	2 copies	2 copies	1 copy	1 copy

2021	422	2 copies	2 copies	1 copy	1 copy
2021	423	2 copies	2 copies	1 copy	1 copy
2021	424	2 copies	2 copies	1 copy	1 copy
2021	425	2 copies	2 copies	1 copy	1 copy
2021	426	2 copies	2 copies	3 copies	3 copies
2021	427	2 copies	2 copies	1 copy	1 copy
2021	428	2 copies	2 copies	1 copy	1 copy
2021	429	2 copies	2 copies	1 copy	1 copy
2021	430	2 copies	2 copies	1 copy	1 copy
2021	431	2 copies	2 copies	1 copy	1 copy
2021	432	2 copies	2 copies	3 copies	3 copies
2021	433	2 copies	2 copies	1 copy	1 copy
2021	434	2 copies	2 copies	1 copy	1 copy
2021	435	2 copies	2 copies	1 copy	1 copy
2021	436	2 copies	2 copies	1 copy	1 copy
2021	437	2 copies	2 copies	1 copy	1 copy
2021	438	2 copies	2 copies	1 copy	1 copy
2021	439	2 copies	2 copies	1 copy	1 copy
2021	440	2 copies	2 copies	3 copies	3 copies
2021	441	2 copies	2 copies	1 copy	1 copy
2021	442	2 copies	2 copies	1 copy	1 copy
2021	443	2 copies	2 copies	3 copies	3 copies
2021	444	2 copies	2 copies	1 copy	1 copy
2021	445	2 copies	2 copies	1 copy	1 copy
2021	446	2 copies	2 copies	1 copy	1 copy

2021	447	2 copies	2 copies	1 copy	1 copy
2021	448	2 copies	2 copies	1 copy	1 copy
2021	449	2 copies	2 copies	1 copy	1 copy
2021	450	2 copies	2 copies	1 copy	1 copy
2021	451	2 copies	2 copies	1 copy	1 copy
2021	452	2 copies	2 copies	3 copies	3 copies
2021	453	2 copies	2 copies	0 copy	0 copy
2021	454	2 copies	2 copies	1 copy	1 copy
2021	455	2 copies	2 copies	1 copy	1 copy
2021	456	2 copies	2 copies	1 copy	1 copy
2021	457	2 copies	2 copies	1 copy	1 copy
2021	458	2 copies	2 copies	1 copy	1 copy
2021	459	2 copies	2 copies	1 copy	1 copy
2021	460	2 copies	2 copies	1 copy	1 copy
2021	461	2 copies	2 copies	1 copy	1 copy
2021	462	2 copies	2 copies	0 copy	0 copy
2021	463	2 copies	2 copies	1 copy	1 copy
2021	464	2 copies	2 copies	1 copy	1 copy
2021	465	2 copies	2 copies	1 copy	1 copy
2021	466	2 copies	2 copies	0 copy	0 copy
2021	467	2 copies	2 copies	1 copy	1 copy
2021	468	2 copies	2 copies	1 copy	1 copy
2021	469	2 copies	2 copies	1 copy	1 copy
2021	706	1 copy	1 copy	1 copy	1 copy
2021	707	1 copy	1 copy	2 copies	2 copies

2021	708	2 copies	2 copies	1 copy	1 copy
2022	470	2 copies	2 copies	3 copies	3 copies
2022	471	1 copy	1 copy	1 copy	1 copy
2022	472	2 copies	2 copies	1 copy	1 copy
2022	473	2 copies	1 copy	2 copies	2 copies
2022	474	2 copies	2 copies	1 copy	1 copy
2022	475	1 copy	1 copy	2 copies	2 copies
2022	476	1 copy	1 copy	2 copies	2 copies
2022	477	1 copy	1 copy	2 copies	2 copies
2022	478	1 copy	1 copy	1 copy	1 copy
2022	479	1 copy	1 copy	2 copies	2 copies
2022	480	1 copy	1 copy	2 copies	2 copies
2022	481	1 copy	1 copy	2 copies	2 copies
2022	482	1 copy	1 copy	2 copies	2 copies
2022	483	1 copy	1 copy	2 copies	2 copies
2022	484	1 copy	1 copy	2 copies	2 copies
2022	485	1 copy	1 copy	2 copies	2 copies
2022	486	1 copy	2 copies	2 copies	1 copy
2022	487	1 copy	1 copy	3 copies	3 copies
2022	488	1 copy	1 copy	1 copy	1 copy
2022	489	1 copy	1 copy	4 copies	4 copies
2022	490	1 copy	2 copies	2 copies	1 copy
2022	491	2 copies	2 copies	1 copy	1 copy
2022	492	1 copy	1 copy	0 copy	0 copy
2022	493	2 copies	1 copy	2 copies	3 copies

2022	494	1 copy	1 copy	2 copies	3 copies
2022	495	1 copy	1 copy	2 copies	3 copies
2022	496	1 copy	1 copy	1 copy	1 copy
2022	497	1 copy	1 copy	3 copies	3 copies
2022	498	1 copy	1 copy	1 copy	1 copy
2022	499	2 copies	1 copy	2 copies	3 copies
2022	500	2 copies	2 copies	0 copy	0 copy
2022	501	2 copies	1 copy	2 copies	3 copies
2022	502	1 copy	2 copies	2 copies	1 copy
2022	503	2 copies	1 copy	2 copies	3 copies
2022	504	2 copies	2 copies	1 copy	1 copy
2022	505	1 copy	1 copy	2 copies	3 copies
2022	506	2 copies	1 copy	2 copies	3 copies
2022	507	1 copy	1 copy	1 copy	1 copy
2022	508	1 copy	1 copy	2 copies	2 copies
2022	509	1 copy	4 copies	3 copies	1 copy
2022	510	1 copy	1 copy	1 copy	1 copy
2022	511	1 copy	1 copy	2 copies	2 copies
2022	512	1 copy	1 copy	2 copies	2 copies
2022	513	3 copies	2 copies	2 copies	2 copies
2022	514	1 copy	1 copy	1 copy	1 copy
2022	515	2 copies	2 copies	0 copy	0 copy
2022	516	1 copy	2 copies	3 copies	2 copies
2022	517	2 copies	2 copies	0 copy	0 copy
2022	518	3 copies	2 copies	2 copies	2 copies

2022	519	1 copy	1 copy	2 copies	2 copies
2022	520	1 copy	1 copy	2 copies	2 copies
2022	521	3 copies	2 copies	2 copies	2 copies
2022	522	3 copies	2 copies	2 copies	2 copies
2022	523	2 copies	1 copy	2 copies	2 copies
2022	524	3 copies	2 copies	2 copies	2 copies
2022	525	3 copies	2 copies	2 copies	2 copies
2022	526	1 copy	2 copies	1 copy	1 copy
2022	527	3 copies	2 copies	2 copies	2 copies
2022	528	2 copies	2 copies	1 copy	1 copy
2022	529	2 copies	1 copy	2 copies	2 copies
2022	530	3 copies	2 copies	1 copy	1 copy
2022	531	2 copies	1 copy	2 copies	2 copies
2022	532	2 copies	2 copies	1 copy	1 copy
2022	533	1 copy	1 copy	1 copy	1 copy
2022	534	1 copy	2 copies	2 copies	2 copies
2022	535	2 copies	1 copy	2 copies	2 copies
2022	536	2 copies	1 copy	2 copies	3 copies
2022	537	1 copy	1 copy	2 copies	2 copies
2022	538	1 copy	1 copy	2 copies	2 copies
2022	539	1 copy	1 copy	2 copies	2 copies
2022	540	1 copy	1 copy	3 copies	3 copies
2022	541	1 copy	1 copy	3 copies	3 copies
2022	542	2 copies	1 copy	2 copies	3 copies
2022	543	2 copies	2 copies	1 copy	1 copy

2022	544	3 copies	3 copies	2 copies	2 copies
2022	545	1 copy	1 copy	2 copies	2 copies
2022	546	2 copies	1 copy	2 copies	3 copies
2022	547	2 copies	1 copy	2 copies	3 copies
2022	548	2 copies	2 copies	1 copy	1 copy
2022	549	2 copies	2 copies	1 copy	1 copy
2022	550	2 copies	1 copy	2 copies	3 copies
2022	551	2 copies	2 copies	0 copy	0 copy
2022	552	2 copies	1 copy	2 copies	3 copies
2022	553	2 copies	2 copies	1 copy	1 copy
2022	554	2 copies	2 copies	1 copy	1 copy
2022	555	2 copies	2 copies	1 copy	1 copy
2022	556	1 copy	2 copies	3 copies	2 copies
2022	557	1 copy	1 copy	2 copies	2 copies
2022	558	1 copy	1 copy	2 copies	2 copies
2022	559	2 copies	2 copies	0 copy	0 copy
2022	560	1 copy	1 copy	2 copies	2 copies
2022	561	2 copies	2 copies	3 copies	3 copies
2022	562	2 copies	1 copy	2 copies	3 copies
2022	563	1 copy	1 copy	3 copies	3 copies
2022	564	2 copies	2 copies	1 copy	1 copy
2022	565	1 copy	1 copy	2 copies	2 copies
2022	566	1 copy	1 copy	1 copy	1 copy
2022	567	2 copies	1 copy	2 copies	3 copies
2022	568	2 copies	2 copies	1 copy	1 copy

2022	569	2 copies	2 copies	1 copy	1 copy
2022	570	2 copies	2 copies	1 copy	1 copy
2022	571	1 copy	1 copy	4 copies	4 copies
2022	572	1 copy	1 copy	1 copy	1 copy
2022	573	1 copy	1 copy	2 copies	2 copies
2022	574	2 copies	2 copies	0 copy	0 copy
2022	575	2 copies	2 copies	0 copy	0 copy
2022	576	2 copies	2 copies	0 copy	0 copy
2022	577	1 copy	1 copy	1 copy	1 copy
2022	578	1 copy	1 copy	1 copy	1 copy
2022	579	1 copy	3 copies	2 copies	2 copies
2022	580	2 copies	2 copies	1 copy	1 copy
2022	581	2 copies	2 copies	1 copy	1 copy
2022	582	2 copies	2 copies	0 copy	0 copy
2022	583	2 copies	2 copies	1 copy	1 copy
2022	584	2 copies	3 copies	2 copies	1 copy
2022	585	1 copy	1 copy	2 copies	2 copies
2022	586	1 copy	1 copy	2 copies	2 copies
2022	587	2 copies	2 copies	0 copy	0 copy
2022	588	1 copy	1 copy	2 copies	2 copies
2022	589	1 copy	1 copy	2 copies	2 copies
2022	590	2 copies	1 copy	2 copies	3 copies
2022	591	1 copy	1 copy	2 copies	2 copies
2022	592	1 copy	1 copy	2 copies	2 copies
2022	593	1 copy	1 copy	3 copies	2 copies

2022	594	2 copies	2 copies	0 copy	0 copy
2022	595	1 copy	1 copy	2 copies	2 copies
2022	596	2 copies	2 copies	1 copy	1 copy
2022	597	3 copies	3 copies	3 copies	2 copies
2022	598	2 copies	2 copies	1 copy	1 copy
2022	599	2 copies	2 copies	1 copy	1 copy
2022	600	2 copies	2 copies	1 copy	1 copy
2022	601	2 copies	2 copies	1 copy	1 copy
2022	602	1 copy	1 copy	2 copies	2 copies
2022	603	2 copies	2 copies	1 copy	1 copy
2022	604	2 copies	2 copies	1 copy	1 copy
2022	605	1 copy	1 copy	1 copy	1 copy
2022	606	3 copies	2 copies	3 copies	2 copies
2022	607	1 copy	1 copy	3 copies	3 copies
2022	608	1 copy	1 copy	2 copies	2 copies
2022	609	2 copies	2 copies	1 copy	1 copy
2022	610	3 copies	2 copies	2 copies	2 copies
2022	611	2 copies	1 copy	2 copies	2 copies
2022	612	3 copies	2 copies	2 copies	2 copies
2022	613	2 copies	2 copies	0 copy	0 copy
2022	614	3 copies	2 copies	1 copy	1 copy
2022	615	3 copies	2 copies	2 copies	2 copies
2022	616	1 copy	1 copy	3 copies	3 copies
2022	617	1 copy	1 copy	2 copies	2 copies
2022	618	1 copy	1 copy	2 copies	2 copies

2022	619	1 copy	1 copy	3 copies	2 copies
2022	620	2 copies	2 copies	0 copy	0 copy
2022	621	2 copies	2 copies	1 copy	1 copy
2022	622	2 copies	2 copies	1 copy	1 copy
2022	623	2 copies	2 copies	1 copy	1 copy
2022	624	2 copies	2 copies	1 copy	1 copy
2022	625	2 copies	2 copies	1 copy	1 copy
2022	626	2 copies	2 copies	1 copy	1 copy
2022	627	1 copy	1 copy	3 copies	3 copies
2022	628	2 copies	2 copies	1 copy	1 copy
2022	629	2 copies	2 copies	1 copy	1 copy
2022	630	2 copies	2 copies	1 copy	1 copy
2022	631	2 copies	2 copies	1 copy	1 copy
2022	632	2 copies	2 copies	1 copy	1 copy
2022	633	2 copies	2 copies	1 copy	1 copy
2022	634	1 copy	1 copy	1 copy	1 copy
2022	635	2 copies	1 copy	1 copy	2 copies
2022	636	1 copy	1 copy	2 copies	2 copies
2022	637	2 copies	2 copies	1 copy	1 copy
2022	638	1 copy	1 copy	2 copies	2 copies
2022	639	2 copies	2 copies	1 copy	1 copy
2022	640	2 copies	2 copies	1 copy	1 copy
2022	641	2 copies	2 copies	1 copy	1 copy
2022	642	2 copies	2 copies	1 copy	1 copy
2022	643	2 copies	2 copies	0 copy	0 copy

2022	644	2 copies	2 copies	1 copy	1 copy
2022	645	2 copies	2 copies	0 copy	0 copy
2022	646	2 copies	3 copies	1 copy	2 copies
2022	647	1 copy	1 copy	2 copies	2 copies
2022	648	1 copy	1 copy	2 copies	2 copies
2022	649	2 copies	2 copies	1 copy	1 copy
2022	650	1 copy	1 copy	2 copies	2 copies
2022	651	2 copies	2 copies	3 copies	3 copies
2022	652	2 copies	1 copy	1 copy	2 copies
2022	709	1 copy	1 copy	3 copies	3 copies
2022	710	1 copy	1 copy	1 copy	1 copy
2022	711	1 copy	2 copies	3 copies	2 copies
2022	712	1 copy	1 copy	1 copy	1 copy
2022	713	1 copy	2 copies	1 copy	1 copy
2022	714	2 copies	1 copy	2 copies	3 copies
2022	715	2 copies	1 copy	2 copies	3 copies
2022	716	1 copy	2 copies	2 copies	1 copy
2022	717	3 copies	3 copies	1 copy	1 copy
2022	718	1 copy	1 copy	3 copies	3 copies
2022	719	2 copies	2 copies	1 copy	1 copy
2023	653	1 copy	1 copy	1 copy	1 copy
2023	654	2 copies	2 copies	1 copy	1 copy
2023	655	2 copies	2 copies	1 copy	1 copy
2023	656	2 copies	2 copies	1 copy	1 copy
2023	657	2 copies	2 copies	1 copy	1 copy

2023	658	2 copies	2 copies	1 copy	1 copy
2023	659	3 copies	3 copies	1 copy	1 copy
2023	660	2 copies	2 copies	1 copy	1 copy
2023	661	2 copies	2 copies	3 copies	3 copies
2023	662	2 copies	2 copies	1 copy	1 copy
2023	663	2 copies	2 copies	0 copy	0 copy
2023	664	2 copies	2 copies	1 copy	1 copy
2023	665	2 copies	2 copies	1 copy	1 copy
2023	666	1 copy	1 copy	2 copies	2 copies
2023	667	2 copies	3 copies	2 copies	1 copy
2023	668	1 copy	1 copy	3 copies	3 copies
2023	669	3 copies	3 copies	0 copy	0 copy
2023	670	2 copies	2 copies	1 copy	1 copy
2023	671	1 copy	1 copy	2 copies	2 copies
2023	672	2 copies	2 copies	1 copy	1 copy
2023	673	1 copy	1 copy	3 copies	3 copies
2023	674	1 copy	1 copy	2 copies	2 copies
2023	675	2 copies	1 copy	2 copies	3 copies
2023	676	2 copies	2 copies	1 copy	1 copy
2023	677	2 copies	2 copies	1 copy	1 copy
2023	678	1 copy	1 copy	2 copies	2 copies
2023	679	1 copy	4 copies	3 copies	1 copy
2023	680	1 copy	1 copy	1 copy	1 copy
2023	681	1 copy	1 copy	2 copies	2 copies
2023	682	1 copy	2 copies	2 copies	1 copy

2023	683	1 copy	1 copy	2 copies	2 copies
2023	684	1 copy	1 copy	3 copies	3 copies
2023	685	1 copy	1 copy	2 copies	2 copies
2023	686	2 copies	1 copy	1 copy	1 copy
2023	687	2 copies	2 copies	0 copy	0 copy
2023	688	1 copy	1 copy	3 copies	3 copies
2023	689	2 copies	2 copies	1 copy	1 copy
2023	690	1 copy	1 copy	3 copies	3 copies
2023	691	1 copy	1 copy	3 copies	3 copies
2023	692	1 copy	1 copy	3 copies	3 copies
2023	693	1 copy	2 copies	3 copies	2 copies
2023	694	1 copy	1 copy	2 copies	2 copies
2023	695	2 copies	1 copy	2 copies	3 copies
2023	696	1 copy	1 copy	3 copies	3 copies
2023	697	2 copies	2 copies	1 copy	1 copy
2023	698	1 copy	1 copy	3 copies	3 copies
2023	699	1 copy	1 copy	2 copies	2 copies
2023	700	2 copies	2 copies	1 copy	1 copy
2023	701	2 copies	1 copy	2 copies	3 copies
2023	702	1 copy	1 copy	3 copies	3 copies
2023	703	1 copy	1 copy	3 copies	3 copies
2023	704	1 copy	1 copy	3 copies	3 copies
2023	705	1 copy	1 copy	3 copies	3 copies
2023	720	1 copy	1 copy	3 copies	3 copies
2023	721	1 copy	1 copy	1 copy	1 copy

2023	722	2 copies	3 copies	2 copies	2 copies
2023	723	1 copy	1 copy	3 copies	3 copies
2023	724	1 copy	1 copy	1 copy	1 copy
2023	725	1 copy	3 copies	3 copies	1 copy
2023	726	2 copies	2 copies	1 copy	1 copy
